# Outcomes of Percutaneous Coronary Intervention Stratified by Body Mass Index

**DOI:** 10.7759/cureus.108544

**Published:** 2026-05-09

**Authors:** Helen Oletu, Moses C Odoeke, Diana C Nweke, Ede Omosumwen, Bismark Oduro, Okechukwu C Erinne, Obinna H Nwachukwu, Olukunle S Omolayo, Evidence E Ohikhuai, Endurance O Evbayekha, Okelue E Okobi, Tamunoinemi Bob-Manuel

**Affiliations:** 1 Medicine and Surgery, University of Benin, Benin City, NGA; 2 Public Health, University of Wolverhampton, Wolverhampton, GBR; 3 Emergency Medicine, Al Ruwaydah General Hospital Saudi Arabia, Riyadh, SAU; 4 Family Medicine, Duchess International Hospital, Ikeja, NGA; 5 Internal Medicine, Richmond Gabriel University, Arnos Vale, VCT; 6 Internal Medicine, University for Development Studies, Tamale, GHA; 7 Epidemiology, University of Texas Health Science Center at Houston, Houston, USA; 8 Internal Medicine, All Saints University School of Medicine, Roseau, DMA; 9 Internal Medicine, Lugansk State Medical University, Luhansk, UKR; 10 Public Health, Jackson State University School of Public Health, Mississippi, USA; 11 Internal Medicine, St. Luke's Hospital, Chesterfield, USA; 12 Family Medicine, IMG Research Academy &amp; Consulting LLC, Homestead, USA; 13 Family Medicine, Larkin Community Hospital Palm Springs Campus, Miami, USA; 14 Family Medicine, Lakeside Medical Center, Belle Glade, USA; 15 Interventional Cardiology, Montefiore Health System, New York City, USA

**Keywords:** body mass index, in-hospital outcomes, obesity, obesity paradox, percutaneous coronary intervention

## Abstract

Background

The relationship between body mass index (BMI) and outcomes after percutaneous coronary intervention (PCI) remains complex. Although obesity is a major risk factor for coronary artery disease, several observational studies have reported comparable or improved outcomes among overweight or obese patients compared with normal-weight patients, a phenomenon described as the “obesity paradox.” Whether this paradox persists when patients are stratified across specific BMI categories, particularly among obesity classes, remains incompletely understood. This study evaluated in-hospital outcomes after PCI across BMI categories using a large national inpatient database.

Methods

The National Inpatient Sample (NIS) 2016-2020 was queried, and those who underwent PCI were identified using International Statistical Classification of Diseases and Related Health Problems, 10^th^ Revision (ICD-10) codes. After excluding underweight patients, the study population was classified into Healthy Weight (HW), Overweight (OW), Obesity Class I (OB1), Obesity Class II (OB2), and Obesity Class III (OB3) based on their BMI: 19.9-24.9 kg/m², 25-29.9 kg/m², 30-34.9 kg/m², 35-39.9 kg/m², and ≥40 kg/m², respectively. The primary outcome was in-hospital mortality. Secondary outcomes were predictors of mortality, including stroke, acute kidney injury (AKI), hemorrhage, hematoma, cardiogenic shock, acute heart failure, and acute respiratory failure (ARF).

Results

There were 58,919 selected PCIs. Of these, 5.1% (3,029) were HW, 9.3% (5,449) were OW, 30.8% (19,895) were OB1, 25.3% (16,650) were OB2, and 30% (18,496) were OB3.

While the HW group showed a higher mortality rate compared to the OB3 group, 2.4 (1.9-2.9, p<0.0001), mortality was less likely in the other subgroups (OW, OB1, and OB2). ARF 9.9 (8.3-11.9, p<0.0001), cardiogenic shock 7.9 (6.7-9.3, p<0.0001), AKI 2.2 (1.9-2.5, p<0.0001), and increasing age 2.4 (1.7-3.6, p = 0.001) were associated with mortality.

Conclusion

This study revealed that, despite the obesity paradox, among the population with a BMI > 30 kg/m², those with a lower BMI may have a lower mortality risk than their severely obese counterparts.

## Introduction

Obesity, a chronic pathological condition, is defined by excessive adipose tissue accumulation in the body. The severity of obesity is commonly quantified using the body mass index (BMI), a metric derived from a mathematical formula that incorporates an individual’s weight and the square of their height, represented as kilograms per square meter (kg/m²) [1-3]. According to established medical guidelines, an individual is classified as obese when their BMI is ≥ 30 kg/m² [1-3]. Worldwide, obesity has tripled since 1975, while in the United States, from 2017 to 2020, 41.9% of the population was obese, with higher prevalence observed among Black individuals compared to other racial groups [3,4]. Nonetheless, BMI alone does not reflect lean muscle mass or the visceral distribution of fat, both of which are important prognostic factors [1-3].

Obesity is one of the strongest modifiable risk factors for cardiovascular diseases (CVDs). Therefore, it is counterintuitive that the said state may confer mortality benefits on individuals who suffer myocardial infarction and undergo percutaneous coronary interventions (PCI) [5,6]. The literature surrounding the protective effects of obesity in individuals with CVD (referred to as the obesity paradox) has sparked considerable controversy [7]. For example, in a study of 25,384 patients undergoing PCI for ST-elevation myocardial infarction (STEMI) in Sweden, after adjustment for age, sex, and other relevant clinical covariates, the previously observed protective association between obesity and one-year mortality following coronary intervention was no longer statistically significant. Notably, individuals in the overweight BMI category demonstrated the lowest adjusted risk of all-cause mortality, whereas underweight patients had the highest mortality risk [7]. However, in previous studies, the obesity group was compared with the non-obese group, often without stratifying this population according to their BMI class. Furthermore, previous studies analyzing the cardiovascular outcomes amongst the BMI classes are scarce [8,9].

A key limitation in the current literature is the lack of granular analyses that stratify PCI outcomes by specific BMI classes, leaving the true relationship between adiposity and post-PCI mortality incompletely understood. This study aimed to analyze a nationally representative database to assess PCI mortality risk by BMI class and to identify factors associated with mortality in this population.

The abstract of this article was presented at the 2023 Transcatheter Cardiovascular Therapeutics annual conference on October 24, 2023 in San Francisco, CA, USA.

## Materials and methods

Study data

We utilized the Nationwide Inpatient Sample (NIS) database from January 1, 2016, to December 31, 2020. Data analysis was conducted from December 15, 2023, to February 4, 2024. The Institutional Review Board determined that this study was exempt from formal review and informed consent requirements because it utilized the NIS, a publicly available and fully de-identified administrative database. The NIS is developed as part of the Healthcare Cost and Utilization Project (HCUP) sponsored by the Agency for Healthcare Research and Quality. The dataset contains no direct patient identifiers, and HCUP enforces strict data use agreements to safeguard patient confidentiality and prevent re-identification. Therefore, the study met federal criteria for exemption from human subjects research oversight. The Strengthening the Reporting of Observational Studies in Epidemiology (STROBE) reporting guideline was followed throughout the study [10].

The NIS is the largest publicly available all-payer inpatient database in the United States and is designed to produce nationally representative estimates of hospitalizations. The NIS uses a stratified sampling design to capture approximately 20% of inpatient discharges from community hospitals across participating states, representing more than 97% of the U.S. population. Each discharge record includes patient demographics, diagnoses, procedures, hospital characteristics, and outcomes. The HCUP provides sampling weights that allow investigators to generate national estimates from the sampled hospitalizations. The NIS has been widely used in cardiovascular outcomes research, including studies evaluating national trends and outcomes following PCI [11]. The NIS has been widely used to evaluate national PCI trends and obesity utilization, disparities, and outcomes [12-15].

Study population

Adult patients aged ≥18 years who underwent PCI between January 1, 2016, and December 31, 2020, were identified using International Statistical Classification of Diseases and Related Health Problems, 10^th^ Revision (ICD-10) procedure codes (Appendix A) [13]. BMI categories were determined using ICD-10-Clinical Modification (ICD-10-CM) diagnosis codes corresponding to BMI classifications recorded during hospitalization, as direct anthropometric measurements are not available in the NIS dataset. Patients were categorized according to ICD-10 codes into Healthy Weight (HW, BMI 19.9-24.9 kg/m²), Overweight (OW, BMI 25-29.9 kg/m²), Obesity Class I (OB1, BMI 30-34.9 kg/m²), Obesity Class II (OB2, BMI 35-39.9 kg/m²), and Obesity Class III (OB3, BMI ≥40 kg/m²). ICD-10 and ICD-10-CM coding definitions were based on standardized coding systems published by the World Health Organization and the Centers for Disease Control and Prevention [13-15]. BMI categories and PCI status were determined using ICD-10-CM diagnosis codes [14] that correspond to BMI classifications, as direct anthropometric measurements are not available in the NIS database. Because the NIS is a discharge-level database and does not contain unique patient identifiers, it is not possible to distinguish between index PCI procedures, repeat revascularizations, or staged interventions. Each hospitalization involving PCI was therefore treated as an independent observation.

Patients classified as underweight (BMI < 18.5 kg/m²) were excluded to reduce confounding related to frailty and chronic illness. Admissions with missing BMI classification codes were also excluded. Because the NIS is a discharge-level database rather than a patient-level dataset, repeat admissions cannot be identified and were treated as independent observations. Comorbid conditions were identified using ICD-10-CM diagnosis codes consistent with HCUP Elixhauser comorbidity definitions and prior validated administrative database studies [15,16].

A key limitation in the current literature is the lack of granular analyses evaluating PCI outcomes across specific BMI categories rather than broadly comparing obese and non-obese patients. This distinction is clinically important because patients with OB1, OB2, and OB3 may differ substantially in age, comorbidity burden, cardiometabolic profile, physiologic reserve, and procedural risk. Therefore, this study aimed to use a nationally representative inpatient database to evaluate the association between BMI category and in-hospital outcomes among adults undergoing PCI, with particular attention to in-hospital mortality and major complications across HW, OW, OB1, OB2, and OB3 groups.

OB3 was selected as the reference category because this study aimed not only to evaluate the obesity paradox but also to examine heterogeneity within elevated BMI groups. Prior studies have often compared obese versus non-obese patients, which may obscure clinically meaningful differences between obesity classes. By using OB3 as the reference group, we were able to assess whether lower BMI categories, including OW, OB1, and OB2, were associated with different outcomes compared with the most severe obesity category. This approach allowed us to explore whether the relationship between BMI and PCI outcomes follows a graded pattern among patients with elevated BMI.

Outcomes

The objective of the study was to investigate the impact of PCI on individuals across various BMI categories, including those with normal weight, overweight, and obesity. The participants were classified into different BMI classes: HW, OW, OB1, OB2, and OB3 based on their BMI as described above in the ‘study population.’ The primary outcome was in-hospital mortality risk. Secondary outcomes were the factors associated with mortality, including periprocedural complications such as stroke, acute kidney injury (AKI), hemorrhage, hematoma, cardiogenic shock, acute heart failure, and acute respiratory failure (ARF).

Statistical analysis

All analyses were performed using weighted NIS data to generate nationally representative estimates. Categorical variables were reported as frequencies and percentages, and continuous variables were reported as means with standard deviations. Baseline characteristics were compared across BMI categories using Pearson’s chi-square test for categorical variables and Student’s t-test or analysis of variance for continuous variables, as appropriate.

Multivariable logistic regression was used to estimate adjusted odds ratios (aORs) and 95% confidence intervals (CIs) for in-hospital mortality and secondary outcomes. OB3 was selected as the reference group because the primary analytic objective was to evaluate whether outcomes differed across lower BMI categories compared with the most severe obesity category, thereby assessing heterogeneity within elevated BMI groups and examining whether risk increased with greater obesity severity. Regression models adjusted for demographic characteristics, socioeconomic factors, hospital characteristics, and clinically relevant comorbidities, including age, sex, race/ethnicity, insurance type, median household income quartile, hospital region, hospital teaching status, diabetes mellitus, chronic kidney disease (CKD), chronic obstructive pulmonary disease (COPD), smoking, coronary artery disease, hypertension, atrial fibrillation, heart failure, hyperlipidemia, hypothyroidism, stroke, and other comorbid conditions.

Admissions with missing BMI classification were excluded because BMI category was the exposure variable of interest. For covariates with missing values, complete-case analysis was performed. A two-sided p-value <0.05 was considered statistically significant. Statistical analysis was performed using SAS 9.4 (SAS Institute Inc., Cary, NC, USA) [17].

## Results

Baseline characteristics

There were 58,919 selected PCIs. Of these, 5.1% (3,029) were HW, 9.3% (5,449) were OW, 30.8% (19,895) were OB1, 25.3% (16,650) were OB2, and 30.4% (18,496) were OB3. Notably, the HW group was significantly older than the OB3 group and had a higher comorbidity burden, including COPD, CKD, stroke, atrial fibrillation, and heart failure (Table 1). However, the groups OW, OB1, OB2, and OB3 had a higher prevalence of hypertension, hypothyroidism, type II diabetes, and hyperlipidemia (Table 1). 

**Table 1:** Baseline characteristics of the study groups CKD: chronic kidney disease; COPD: chronic obstructive pulmonary disease; ICD: implantable cardioverter defibrillators

Variable	Healthy weight % (n)	Overweight % (n)	Obesity class I % (n)	Obesity class II % (n)	Obesity class III % (n)
Sex					
Female	46% (1,392)	31.8% (1,729)	30.6% (6,086)	36% (5,962)	46.9% (8,659)
Male	54% (1,635)	68.2% (3,707)	69.4% (13,802)	64% (10,600)	53.1% (9,801)
Race					
White	70.5% (2,071)	71.7% (3,787)	75.8% (14,600)	76.4% (12,280)	75.9% (13,556)
Black	13.8% (404)	10% (527)	9.8% (1,885)	10.9% (1,744)	13.7% (2,452)
Hispanic	7.1% (208)	11.2% (589)	9.1% (1,748)	8.3% (1,336)	6.8% (1,208)
Asian/Pacific Islander	3.9% (114)	2.9% (151)	1.6% (312)	1.2% (185)	0.8% (137)
Mean age, years	68.5 ±10.7	64 ±11.2	62.3 ±11.2	60.7 ±11.3	59.4 ±11.4
Length of hospitalization, days	7.8 ±9.1	4.3 ±5.2	3.5 ±3.8	3.6 ±3.6	4.5 ±5.3
Insurance type					
Medicare	67.5% (2,044)	50.2% (2,733)	45.7% (9,076)	43.2% (7,178)	44.8% (8,278)
Medicaid	11.5% (349)	10.1% (549)	9.9% (1,961)	11.2% (1,858)	13.1% (2,421)
Private insurance	14.6% (442)	30.4% (1,654)	35.3% (7,010)	36.8% (6,116)	33.6% (6,203)
Self-pay	3.1% (94)	5.4% (295)	5.1% (1,017)	5.1% (855)	5.1% (950)
Others	2.8% (85)	3.2% (175)	3.5% (690)	3.2% (539)	2.9% (543)
Median household income					
0–25^th^ percentile	34.6% (1,021)	30% (1,607)	28.9% (5,597)	29.9% (4,918)	33% (6,020)
26–50^th^ percentile	26.8% (791)	27.2% (1,453)	27.9% (5,466)	27.9% (4,585)	29.5% (5,364)
51–75^th^ percentile	22.6% (668)	23.9% (1,278)	24.4% (4,790)	24.8% (4,067)	23.6% (4,295)
76–100^th^ percentile	16.1% (475)	18.9% (1,014)	19.1% (3,752)	17.3% (2,834)	13.9% (2,536)
Region of the hospital					
Northeast	15.7% (476)	14.7% (799)	16.3% (3,243)	15.7% (2,615)	15.5% (2,872)
Midwest	27% (818)	22.7% (1,234)	27.3% (5,427)	28.2% (4,700)	28.7% (5,312)
South	40.1% (1,215)	43.6% (2,378)	39.4% (7,843)	40% (6,646)	42.2% (7,796)
West	17.1% (520)	19.1% (1,038)	17% (3,382)	16.2% (2,689)	13.6% (2,516)
Location/Teaching status					
Rural	6% (181)	5.3% (288)	5.2% (1,029)	5.8% (962)	6.5% (1,212)
Urban nonteaching	19.4% (588)	21.6% (1,175)	20.2% (4,007)	21% (3,497)	21% (3,870)
Urban teaching	74.6% (2,260)	73.1% (3,983)	76.6% (14,851)	73.2% (12,161)	72.5% (13,378)
Comorbidities					
Type II diabetes	8.9% (271)	19.6% (1,068)	21% (4,176)	23.5% (3,907)	23.8% (4,398)
CKD	27.6% (837)	20% (1,091)	18.9% (3,771)	20.1% (3,334)	24.5% (4,521)
COPD	37.2% (1,128)	15.4% (838)	14.7% (2,913)	15.4% (2,565)	19.5% (3,595)
Chronic steroid use	2.6% (80)	1.2% (66)	0.9% (177)	0.9% (152)	0.9% (164)
Smoking	52.4% (1,588)	48.9% (2,664)	49.1% (9,772)	48.4% (8,040)	43.5% (8,030)
Cannabis	3.1% (93)	2.3% (123)	1.9% (375)	1.7% (290)	1.6% (295)
ICD	2.1% (64)	1.7% (90)	1.2% (237)	1.3% (218)	1.6% (288)
Stroke	1.9% (59)	1.1% (62)	0.7% (129)	0.6% (107)	0.7% (136)
Hypothyroidism	10.7% (323)	9.6% (525)	9.8% (1,955)	11.4% (1,890)	13.9% (2,573)
Coronary artery disease	86.3% (2,614)	89.6% (4,877)	90.2% (17,947)	90.2% (14,990)	88.4% (16,317)
Hypertension	77.3% (2,340)	84.5% (4,602)	86.6% (17,226)	89.3% (14,835)	90.1% (16,625)
Supraventricular tachycardia	3.9% (119)	2.1% (112)	1.8% (350)	1.6% (270)	1.7% (317)
Atrial fibrillation	19.5% (592)	12.6% (685)	13.3% (2,641)	13.4% (2,230)	15.8% (2,920)
Ventricular fibrillation	4.3% (131)	3.5% (189)	3.2% (634)	2.7% (455)	3.4% (623)
Heart failure	47.5% (1,438)	28.5% (1,551)	26.6% (5,289)	29.4% (4,888)	38% (7,013)
Pulmonary embolism	0.86% (26)	0.44% (24)	0.30% (60)	0.25% (42)	0.45% (83)
Hyperlipidemia	53.8% (1,628)	73.7% (4,012)	74.4% (14,793)	73.8% (12,266)	70% (12,914)
Major depressive disorder	12.5% (377)	9.7% (526)	10.2% (2,026)	10.9% (1,824)	12.9% (2,395)
Bipolar disorder	1.5% (46)	1.1% (62)	1.3% (261)	1.4% (233)	1.9% (361)
Generalized anxiety disorder	16.7% (506)	12.4% (676)	12.2% (2,433)	12.2% (2,024)	12.9% (2,381)

Outcomes

Although the HW subgroup showed a higher likelihood of mortality compared to the OB3 subgroup, with an OR of 2.4 (1.9-2.9, p<0.0001), mortality was less likely in the other subgroups (OW, OB1, and OB2) as indicated in Table 2.

**Table 2:** Outcomes table with OB3 as the reference category. Values are represented as odds ratios with their confidence intervals. OB1: Obesity Class I; OB2: Obesity Class II; OB3: Obesity Class III; HW: Healthy Weight; OW: Overweight

Primary and secondary outcomes				
	HW vs. OB3	OW vs. OB3	OB1 vs. OB3	OB2 vs. OB3
Mortality	2.4 (1.9-2.9, p<0.0001)	0.6 (0.4-0.8, p<0.0001)	0.6 (0.5-0.7, p<0.0001)	0.7 (0.6-0.9, p<0.0001)
Postoperative hemorrhage	0.9 (0.6-1.8, p=0.7)	0.8 (0.5-1.5, p=0.9)	0.7 (0.4-1.0, p=0.2)	0.8 (0.5-1.2, p=0.7)
Postoperative hematoma	0.6 (0.3-0.9, p=0.1)	1.0 (0.7-1.5, p=0.2)	0.8 (0.6-1.0, p=0.9)	0.9 (0.7-1.2, p=0.3)
Acute respiratory failure	1.7 (1.5-1.9, p<0.0001)	0.7 (0.6-0.8, p=0.08)	0.5 (0.4-0.6, p<0.0001)	0.6 (0.5-0.6, p<0.0001)
Acute kidney injury	1.2 (1.1-1.4, p<0.0001)	0.8 (0.7-0.8, p=0.01)	0.7 (0.6-0.7, p<0.0001)	0.7 (0.6-0.8, p<0.0001)
Postoperative cardiac shock	2.1 (1.8-2.4, p<0.0001)	1.3 (1.2-1.5, p=0.002)	0.9 (0.9-1.1, p=0.1)	0.8 (0.7-0.9, p<0.0001)
Cardiac arrest	1.4 (1.1-1.7, p<0.0001)	0.9 (0.7-1.2, p=0.8)	0.8 (0.7-0.9, p=0.001)	0.8 (0.7-0.9, p=0.001)
Acute heart failure	0.6 (0.1-6.0, p=0.9)	0.9 (0.2-4.0, p=0.8)	0.6 (0.2-1.9, p=0.7)	0.7 (0.2-2.3, p=0.9)
Stroke	2.3 (1.6-3.3, p<0.0001)	1.6 (1.2-2.1, p=0.003)	1 (0.8-1.3, p=0.09)	0.9 (0.8-1.4, p=0.2)

Additionally, ARF, cardiogenic shock, AKI, and increasing age were independently associated with mortality, with ORs of 9.9 (8.3-11.9, p<0.0001), 7.9 (6.7-9.3, p<0.0001), 2.2 (1.9-2.5, p<0.0001), and 2.4 (1.7-3.6, p=0.001), respectively.

## Discussion

**Figure 1:**
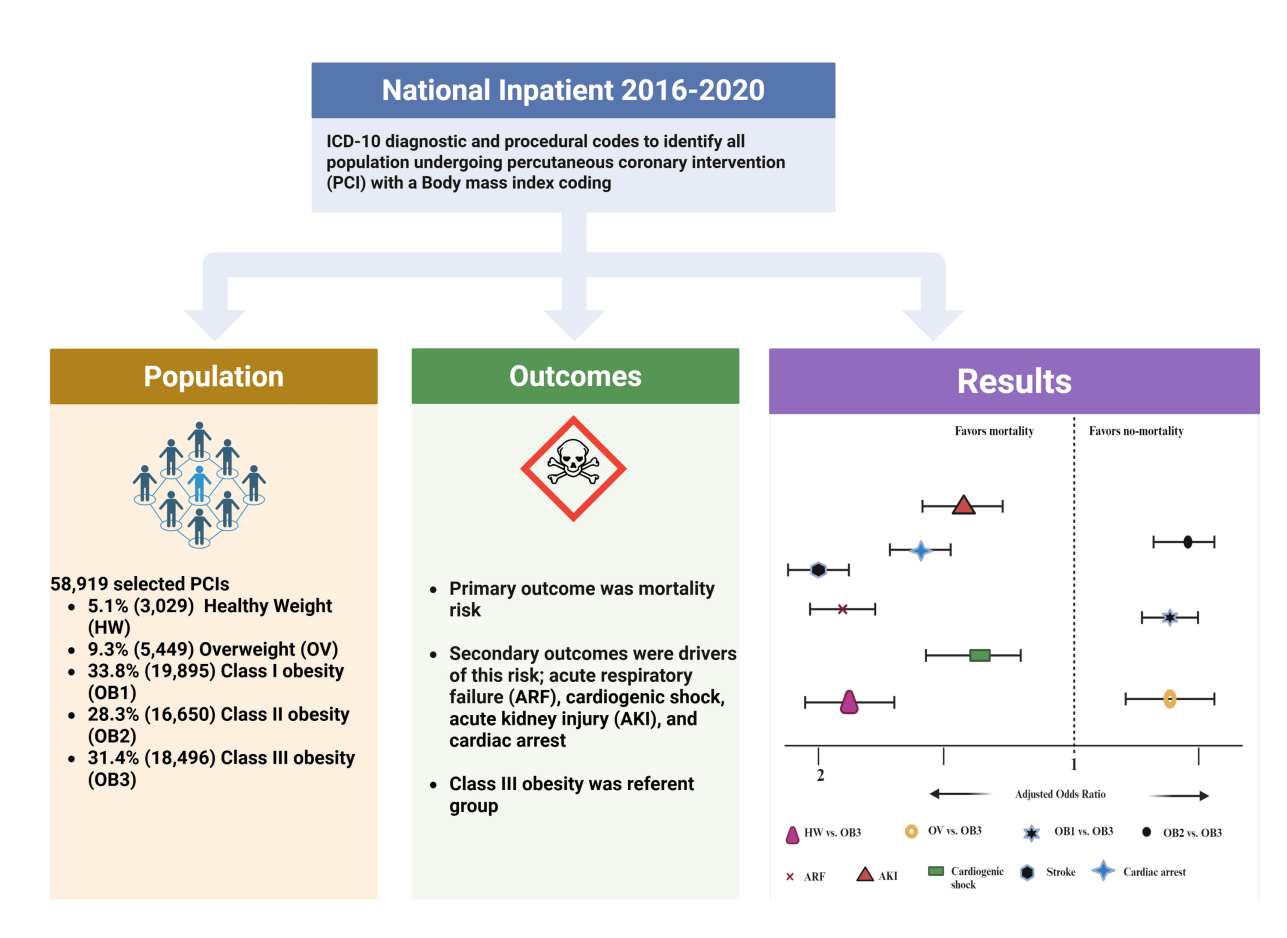
Methodology, outcomes, and results captured ICD-10: International Classification of Diseases, 10^th^ Revision Original illustration by Evbayekha et al., created using BioRender.com.

In this nationally representative analysis of PCI hospitalizations stratified by BMI category, we observed several important findings (Figure 1). First, HW patients had higher odds of in-hospital mortality compared with patients with OB3. Second, among patients with elevated BMI, the OW, OB1, and OB2 groups had lower odds of mortality compared with the OB3 group. Third, the HW group was substantially older and had a higher prevalence of several high-risk comorbidities, including CKD, COPD, atrial fibrillation, stroke, and heart failure. Fourth, ARF, cardiogenic shock, AKI, and increasing age were independently associated with mortality.

This study's findings indicate that the HW group had a higher likelihood of in-hospital mortality (2.4; 1.9-2.9; p < 0.00001) compared to the OB3 group. However, there was a lower likelihood of in-hospital mortality in the OW (0.6; 0.4-0.8; p < 0.0001), OB1 (0.6; 0.5-0.7; p < 0.0001), and OB2 (0.7; 0.6-0.9; p < 0.0001) groups when compared to the OB3 group. These results demonstrate that, despite the observed obesity paradox between the HW and OB3 groups, the lower BMI subgroups showed a lower in-hospital mortality rate than their obese counterparts among the overweight and obese subgroups. This is consistent with a retrospective study conducted by Kanic et al. in their analysis of 6,496 patients [18]. The studied population was categorized into six BMI groups, and they found that those in the OB1 group had the lowest mortality rate. Consequently, those with a BMI < 25 kg/m^2^ had a higher mortality risk. It is noteworthy that underweight patients had a two-fold increase in mortality, and OB3 was associated with over 70% long-term mortality risk [18]. The study demonstrated the obesity paradox but also highlighted that both lower and higher BMI can be harmful to patients with myocardial infarction who undergo PCI [18]. The complexity of BMI-related mortality risks emphasizes the need for nuanced considerations.

Interestingly, Reinstadler et al. compared myocardial infarct size using cardiac magnetic resonance between BMI <25 kg/m^2^, 25-30 kg/m^2^, and >30 kg/m^2^ and found no correlation between BMI and infarct size or extent of microvascular obstruction [19]. They, however, saw a significantly lower major adverse cardiac event-free survival at 12 months in the <25 kg/m^2^ BMI group [19]. Another study demonstrated less favorable survival outcomes in acute myocardial infarction in malnourished nonobese individuals than in malnourished obese individuals when compared with the nourished nonobese population. They noted that the malnourished nonobese population had the highest all-cause mortality [20]. Sinjini et al. conducted a multicenter study involving 25,413 patients who underwent PCI, further corroborating our findings that a mildly elevated BMI of < 35 kg/m2 had less mortality likelihood than the severely elevated BMI (OB3) group [21].

Of the factors associated with in-hospital mortality, ARF had one of the strongest associations. Previous research has emphasized the impact of ARF on PCI outcomes [22]. Individuals who experience ARF during or after PCI face significantly elevated mortality risk. Hypoxemia leads to inadequate oxygen exchange, exacerbating myocardial ischemia and compromising tissue viability. Increased cardiac workload is another mechanism by which ARF strains the cardiovascular system, especially in obese patients with pre-existing cardiac conditions [23,24]. The inflammatory response is the third mechanism of ARF, which triggers systemic inflammation, contributing to endothelial dysfunction and plaque instability [24,25]. 

Postoperative cardiogenic shock was also associated with in-hospital mortality in this study. Buschur et al. concluded that mortality in the population with extreme obesity was increasing [26]. They also noted increased mortality in the group with extreme obesity, with cardiogenic shock contributing significantly to mortality [26]. A few studies that specifically paid attention to cardiogenic shock in the population with obesity noted that although the incidence was low, it was associated with a high mortality rate [27,28]. The plausible mechanisms include severe myocardial dysfunction, leading to inadequate tissue perfusion. This culminates in multi-organ failure from reduced cardiac output [29,30]. 

Furthermore, AKI, stroke, and increasing age were all independently associated with increased likelihood of in-hospital mortality. Many studies have correlated these findings [22,31-33]. Additionally, other studies noted an inflammatory response as one of the mechanisms of mortality in AKI, impacting the overall prognosis [32,34]. Chen et al. noted that age remains an immutable risk factor for mortality in those undergoing PCI [35]. This may be due to increased comorbidity burden and declining physiological reserves, impacting their ability to tolerate stressors. This is supported by the analysis in this study, which found the HW population to be older and to have a higher comorbidity prevalence.

This study delved into potential explanations for the obesity paradox. First, age may have played a significant role in these results. The OB3 group (59.4 ± 11.4), which had a lower in-hospital mortality rate compared to the HW group (68.5 ± 10.7), was notably younger (mean age difference = 9.1 years). This highlights the potential influence of age on the obesity paradox. Younger patients have more significant physiological reserves to tolerate and compensate for adverse medical conditions, leading to better outcomes than their older counterparts [34,36]. We also noted a higher prevalence of comorbidities such as CKD, COPD, and implantable cardioverter defibrillators in the HW group, suggesting a complex interplay of age and comorbidities. In addition, medications commonly prescribed to patients with a higher BMI, such as statins and aspirin, along with lifestyle modifications, including diet, exercise, and smoking cessation, may contribute to the observed paradox [37].

Limitations 

This study has several limitations. First, the NIS is an administrative database, and diagnoses, procedures, BMI categories, comorbidities, and outcomes are identified using ICD-10 codes. Therefore, coding errors, misclassification, and underreporting are possible. Second, BMI categories were determined using diagnosis codes rather than direct height and weight measurements, as anthropometric measurements are not available in the NIS. Third, admissions without BMI classification codes were excluded, which may introduce selection bias if BMI coding was more likely among certain patient groups. Fourth, although multivariable adjustment was performed, residual confounding remains possible. The NIS does not capture several clinically important variables, including frailty, nutritional status, sarcopenia, functional capacity, body composition, visceral adiposity, medication use, procedural complexity, coronary anatomy, stent characteristics, access site, laboratory values, left ventricular ejection fraction, and severity of acute coronary syndrome. These unmeasured factors may partly explain the observed association between BMI category and mortality. Fifth, because the NIS is a discharge-level database, repeat admissions cannot be identified, and long-term outcomes after discharge cannot be assessed. Finally, the observational design of this study precludes causal inference. Furthermore, because the NIS is a discharge-level database, repeat hospitalizations for the same patient cannot be identified. As a result, we were unable to differentiate between first-time PCI procedures, repeat interventions, or staged revascularization procedures, which may influence outcomes. Therefore, the findings should be interpreted as associations rather than evidence that the BMI category directly causes differences in PCI outcomes.

## Conclusions

In this large, nationally representative analysis of PCI hospitalizations. HW patients had higher observed odds of in-hospital mortality compared with patients with OB3; however, this finding may be influenced by older age, higher comorbidity burden, frailty, nutritional status, and residual confounding. Among patients with elevated BMI, OW, OB1, and OB2 were associated with lower odds of mortality compared with OB3, suggesting heterogeneity in risk across obesity classes. These findings support a more nuanced interpretation of the obesity paradox after PCI and highlight the need for future studies incorporating body composition, frailty, medication use, procedural characteristics, and long-term outcomes.

## Appendices

Appendix A 

**Table 3:** ICD-10 order and procedural codes STEMI: ST-segment elevation myocardial infarction; NSTEMI: Non-STEMI; ICD: intracardiac defibrillator; ECMO: extracorporeal membrane oxygenation; ICD-10: International Classification of Diseases, 10^th^ Revision Source: [13]

International Classification of Disease Tenth Edition (ICD-10) Codes	Diagnosis/Procedure
COVID	U071
Hypertension	I10, I11, I119
Hyperlipidemia	E785, E7849, E782, E784
Diabetes mellitus	E11, E119
Myocardial Infarction	
STEMI	I21, I2111, I219, I210, I2119, I219, I2101, I212, I21A9, I2102, I2121, I22, I2109, I2129, I220, I211, I213, I221, I228, I229
NSTEMI	I214, I222
Chronic kidney disease	N18, N181, N182, N183, N1830, N1831, N1832, N184, N185, N186, N189
Obesity	E663, E668, E669, E6601, E6609, E660, E66
Smoking	F17, F203, F17213, F172, F208, F17218, F1720, F209, F17219, F1200, F1721, F1722, F1201, F17210, F17220, F202, F17211, F17221, F17223, F17228, F17229, F1729, F17290, F17291, F17293, F17298, F17299, Z87891
Ventricular fibrillation	I490, I4901, I4902
Supraventricular tachycardia	I471
Sick sinus syndrome	I495
Atrial fibrillation	I48, I480, I481, I4811, I4819, I482, I4820, I4821, I489, I4891
Atrioventricular blocks	
Type 1 block	I440
Type 2 block	I441
Type 3 block	I442
In-hospital outcomes	
Acute respiratory failure	J96, J960, J9600, J9600, J9601, J9602, J961, J9610, J9611, J9612, J962, J9620, J9621, J9622, J969, J9690, J9691, J9692
Pulmonary hypertension	I270, I272, I2720, I2721, I2722, I2723, I2724, I2729
Cardiac arrest	I46, I462, I468, I469
ICD placement	02H60JZ, 02H60KZ, 02H64KZ, 02H70MZ, 02H70NZ, 02H74JZ, 02H74KZ, 02HK3JZ, 02H63JZ, 02H64MZ, 02H70KZ, 02H73JZ, 02H73NZ, 02H74MZ, 02HK0NZ, 02H63KZ, 02H64NZ, 02H70JZ, 02H73KZ, 02H73MZ, 02H74NZ, 02HK0MZ, 02H60NZ, 02H60MZ, 02H63MZ, 02H63NZ, 02H64JZ, 02HK0JZ, 02HK0KZ, 02HK3KZ, 02HK3MZ, 02HK3NZ, 02HK4JZ, 02HK4KZ, 02HK4MZ, 02HK4NZ, 02HL0JZ, 02HL0KZ, 02HL0MZ, 02HL0NZ, 02HL3JZ, 02HL3KZ, 02HL3MZ, 02HL3NZ, 02HL4JZ, 02HL4KZ, 02HL4MZ, 02HL4NZ
Cardiogenic shock	R570, T8111XA, T8111XS
Pulmonary embolism	I2602, I2609, I2692, I2699
Acute right heart failure	I50811, I50813
Invasive mechanical ventilation	0BH17EZ, 0BH18EZ, 5A1935Z, 5A1945Z, 5A1955Z
ECMO	5A1522G, 5A1522H, 5A15A2F, 5A15A2G, 5A15A2H
